# Effect of hospital-at-home vs. traditional brick-and-mortar hospital care in acutely ill adults: study protocol for a pragmatic randomized controlled trial

**DOI:** 10.1186/s13063-022-06430-6

**Published:** 2022-06-16

**Authors:** Xiaoxi Yao, Margaret Paulson, Michael J. Maniaci, Ajani N. Dunn, Chad R. Nelson, Emma M. Behnken, Melissa S. Hart, Lindsey R. Sangaralingham, Shealeigh A. Inselman, Michelle A. Lampman, Shannon M. Dunlay, Sean C. Dowdy, Elizabeth B. Habermann

**Affiliations:** 1 Robert D. and Patricia E. Kern Center for the Science of Health Care Delivery, Mayo Clinic, 200 First Street SW, Rochester, MN 55905 USA; 2 Department of Cardiovascular Medicine, Mayo Clinic, Rochester, MN USA; 3 Division of Hospital Internal Medicine, Mayo Clinic Health Systems, Eau Claire, WI USA; 4 Division of Hospital Internal Medicine, Mayo Clinic, Jacksonville, FL USA; 5 Administrative Operations, Mayo Clinic, Jacksonville, FL USA; 6 Division of Hospital Internal Medicine, Mayo Clinic, Phoenix, AZ USA; 7 Knowledge and Evaluation Research Unit, Mayo Clinic, Rochester, MN USA

**Keywords:** Advanced care at home, Hospital at home, Inpatient, Pragmatic trial

## Abstract

**Background:**

Delivering acute hospital care to patients at home might reduce costs and improve patient experience. Mayo Clinic’s Advanced Care at Home (ACH) program is a novel virtual hybrid model of “Hospital at Home.” This pragmatic randomized controlled non-inferiority trial aims to compare two acute care delivery models: ACH vs. traditional brick-and-mortar hospital care in acutely ill patients.

**Methods:**

We aim to enroll 360 acutely ill adult patients (≥18 years) who are admitted to three hospitals in Arizona, Florida, and Wisconsin, two of which are academic medical centers and one is a community-based practice. The eligibility criteria will follow what is used in routine practice determined by local clinical teams, including clinical stability, social stability, health insurance plans, and zip codes. Patients will be randomized 1:1 to ACH or traditional inpatient care, stratified by site. The primary outcome is a composite outcome of all-cause mortality and 30-day readmission. Secondary outcomes include individual outcomes in the composite endpoint, fall with injury, medication errors, emergency room visit, transfer to intensive care unit (ICU), cost, the number of days alive out of hospital, and patient-reported quality of life. A mixed-methods study will be conducted with patients, clinicians, and other staff to investigate their experience.

**Discussion:**

The pragmatic trial will examine a novel virtual hybrid model for delivering high-acuity medical care at home. The findings will inform patient selection and future large-scale implementation.

**Trial registration:**

ClinicalTrials.gov NCT05212077. Registered on 27 January 2022

**Supplementary Information:**

The online version contains supplementary material available at 10.1186/s13063-022-06430-6.

## Background

Hospital care accounts for one-third of the US medical expenditures yet can be unsafe for some patients, with adverse events being common [1]. Furthermore, many patients prefer to be at home and days spent at home has been increasingly considered a patient-centered goal and outcome [2]. The “Hospital at Home” (HaH) model has been developed to deliver the necessary services, technology, and capabilities to safely shift hospital-level care to a patient’s home. Previous studies suggested that this approach might reduce cost and healthcare use while improving patient outcomes and experience when compared with usual hospital care for acutely ill adults [3–9]. However, these studies are either non-randomized studies or small randomized controlled trials (RCTs), mostly from other countries [10–12].

More importantly, previous HaH programs often are confined to a small number of local patients since most programs rely on clinicians to frequently travel to patients’ homes to deliver care. This model faces scalability barriers as it would be resource-intensive to scale to large, broad populations. Mayo Clinic recently established a novel HaH program, i.e., Advanced Care at Home (ACH), which combines a virtual physician-staffed command center with a vendor-mediated supply chain that can deliver high-acuity care across rural, suburban, and urban settings. In this new model, all physician visits are virtual; the nursing care, including care coordination and planning, is also virtual and synchronized with other care team members who visit patients’ homes. As such, we aim to conduct a pragmatic randomized controlled trial (RCT) to compare two acute care delivery models: ACH vs. traditional brick-and-mortar hospital care in acutely ill patients.

## Methods/design

This is a pragmatic non-inferiority RCT with two parallel groups and 1:1 allocation ratio. A total of 360 acutely ill patients will be enrolled and randomized to either ACH or traditional brick-and-mortar hospital care. The study was approved by the Mayo Clinic Institutional Review Board (IRB) and was registered on ClinicalTrials.gov (NCT05212077). The SPIRIT checklist is attached as Additional file 1 and the World Health Organization Trial Registration Dataset is attached as Additional file 2.

### Setting

The trial will be conducted at Mayo Clinic sites where ACH is currently being delivered, including three hospitals in Phoenix, Arizona, Jacksonville, Florida, and Eau Claire, Wisconsin, respectively. The hospitals in Arizona and Florida are academic medical centers, and the hospital in Wisconsin is a community-based practice.

### Study population

Adult patients (≥18 years) who are acutely ill will be screened for eligibility. Acutely ill patients are those deemed by the emergency department (ED) or inpatient attending physicians as requiring acute inpatient care based on InterQual ® criteria, a well-established set of criteria adopted by most hospitals. Because we aim to compare two care models currently used in routine practice, the local clinical teams (not the research team) will make the final decision regarding eligibility and will refer eligible patients to the research team. The enrollment workflow will follow these steps: if the ED or inpatient attending physician decides to admit or transfer a patient, the physician will contact the ACH team to screen the patient based on clinical criteria, zip code, and health insurance plan. Eligible patients must have a chief complaint currently targeted by the ACH program (Table 1). The patients will be further screened based on the zip codes of their home address since they must reside in areas where ACH can be delivered (Table 2). Patients will also need to have health insurance plans that cover the ACH services (Table 3). In preliminary analyses, most patients were eligible for ACH services based on health insurance plans, as the study population consists of mostly older adults insured by Medicare. Patients randomized to receive either ACH or traditional inpatient hospital care will be reimbursed by their health insurance plans. Although patients might be responsible for certain out-of-pocket costs, such as deductible, copay, and coinsurance, the out-of-pocket costs have been comparable for patients receiving ACH or traditional brick-and-mortar hospital care based on our experience over the past several years. As the study aims to compare two care models already used in routine practice, the research team will not cover the costs of routine care.

**Table 1:** Inclusion criteria—examples of qualifying diagnoses

**Diagnoses included in filter**	
Pneumonia, bronchitis with asthma	
Respiratory failure	
Urinary tract infection (UTI)	
Heart failure (HF)	
Chronic obstructive pulmonary disease (COPD)	
Pulmonary embolism	
Migraines, headaches, syncope, fever	
Gastroenteritis, pancreatitis	
Cellulitis	
Hypovolemia/electrolyte disorders	
Renal failure	
Sepsis, infections, osteomyelitis	
Infectious tendonitis/bursitis and autoimmune myositis	
Disorders of liver/biliary tract	
Re-feeding syndrome/peritoneal infection (non-dialysis)	
Pleural effusion (dependent on primary diagnosis)	
Respiratory illnesses (interstitial lung disease [ILD], respiratory neoplasms)	
Hypertensive emergency	
Inflammatory bowel disease (IBD; with good functional status/mobile)	
Urinary stones	

These are examples for qualifying diagnoses; not every patient with these diagnoses is eligible, and additional diagnoses might be considered; the clinical teams will make the decision, i.e., identifying patients at clinical equipoise who can benefit from either ACH or traditional inpatient care, and will refer eligible patients to the study coordinator for consent and randomization

**Table 2:** Inclusion criteria—zip codes

State	Zip code
Arizona	85054 85050 85254 85032 85024 85260 85266 85022 85028 85255 85027 85023 85258 85253 85377 85020 85327 85085 85259 85029 85053 85331 85250 85021 85016 85083 85269 85271 85267 85252 85261 85018 85310 85251 85268 85051 85014 85308 85306 85012 85013 85304 85015 85008 85086 85302
Florida	3223332260 3222532004 3226632073 3224632095 3222432202 3225032203 3225732204 3225632205 3221632206 3221132208 3227732209 3221732210 3222332218 3208132235 3208232239 3220732240 3222732244 3225932247 3208132254 3225832255
Wisconsin	5470154765 5470254737 5470354736 5472054772 5472254743 5472454757 5472754768 5472954741 5473054747 5473854725 5473954763 5474254749 5475154760 5475554773 5477054728 5477454721 5476454740 5474854771 5473554745 5475854610 5472654756 5473254493

These are zip codes used at the time of IRB submission; not every patient in these zip codes is eligible, and additional zip codes might be considered; the local clinical teams will make the decision, i.e., identifying patients who can benefit from either ACH or traditional inpatient care, and will refer eligible patients to the study coordinator

**Table 3:** Inclusion criteria—health insurance plans

State	Health insurance plan
Arizona	1. Medica—employees only; no midnight rule 2. Medicare—no midnight rule
Florida	1. Medica—employees only; no midnight rule 2. Florida Blue—blue options plan only; no midnight rule 3. Aetna—all Aetna contracted plans; 1 midnight rule 4. Medicare—no midnight rule
Wisconsin	1. Medicare ACO—no midnight rule 2. Medicare—2 midnight rule Removed for CMS waiver go-live on 4/27 3. Medica—employees only; no midnight rule 4. WEA—1 midnight rule 5. Health Tradition—1 midnight rule 6. Security Health Plan—1 midnight rule 7. Anthem—1 midnight rule

*ACO*, accountable care organization; *CMS*, Centers for Medicare & Medicaid Services

These are the health plans used at the time of IRB submission; not every patient with these health plans is eligible, and additional health plans might be considered; the local clinical teams will make the decision, i.e., identifying patients who are suitable for either ACH or traditional inpatient care, and will refer eligible patients to the study coordinator for consent and randomization

If the local clinical team concludes a patient would benefit more from one treatment, then the patient would not be eligible for randomization. Examples of exclusion criteria include residing in a nursing home, on or requiring dialysis, positive for COVID-19, having a discharge order, requiring intensive care unit (ICU) level of care, or a history of drug abuse (Table 4). Patients will also need to meet the clinical stability criteria (Additional file 3: Clinical Stability Screen). Additionally, eligible patients must have the capacity to consent or could assent with the consent of a health care proxy who is physically present.

**Table 4:** Exclusion criteria

**Exclusion criteria**	
Nursing home patient	
Have an order for hemodialysis or peritoneal dialysis and/or listed on the patient’s problem list.	
Positive for COVID-19	
Have a discharge order	
Require intensive care unit (ICU) level of care	
A history of drug abuse	

These are examples of exclusions criteria; the final decision will be made by the local clinical teams (not the research team) to identify patients who can benefit from either ACH or traditional inpatient hospital care (i.e., clinical equipoise) based on the criteria and the judgment used in routine practice. If a patient is judged by local clinical teams not suitable for ACH or traditional inpatient hospital care, the patient cannot be randomized

If a patient is eligible, an ACH case manager or nurse will give a brief introduction of the ACH model to the patient. If the patient is interested, the case manager/nurse will complete the social stability screening (Additional file 4: Social Stability Screening) with them to make a final determination as to whether or not the patient is eligible for ACH. If the patient passes the social stability screening and is still interested in exploring the ACH option, the local clinical team will connect with a study coordinator who will inform patients in more detail of the opportunity to participate in the study, including the study purpose, procedures, risks, and benefits. Potential participants will be informed that participation in the research study is voluntary and that they are free to decline to be in the study or to withdraw from it at any point without any negative consequence. Additionally, they will be informed that the option to receive ACH or traditional brick-and-mortar hospital care will still be available to them outside of participation in the study. The study coordinators will consent and enroll patients using the attached consent form (Additional file 5).

### Randomization

Patients will be randomized in a 1:1 ratio to either ACH or traditional brick-and-mortar hospital care. The randomization algorithm will be generated within the Remote Data Capture (REDCap) software system, using stratified block randomization with blocks of random size. As the clinical trial will be stratified by site, randomization is then performed separately within each stratum level, utilizing blocks of random size. The study coordinators will implement the randomization in REDCap and assign participants. Due to the nature of the intervention, blinding is not feasible for trial participants and care providers. Of the study team members, the study coordinators, analysts, and the data manager will know the randomization due to their roles and responsibilities, including implementing randomization, ensuring data quality, and providing reports to the Data and Safety Monitoring Board (DSMB). The rest of the study team, e.g., the principal investigators and co-investigators, will be blinded to the allocation.

### Intervention

The Mayo Clinic ACH model was previously described (Paulson et al., in press) and the procedures used in the trial will be the same as those established in current routine practice. In short, the ACH program consists of three key components, linked by Medically Home’s Cesia Continuum^TM^ software platform: (1) Command Center, staffed with physicians, registered nurses (RNs), and advanced practice providers (APPs, i.e., physician assistants and advanced nurse practitioners); (2) technology in the home with custom technology kits, including biometric devices for monitoring vital signs, a custom-configured tablet with video visit capability, a telephone to facilitate 2-way communication, a backup power supply, a backup cellular communication device, and an emergency response system bracelet to keep patients and their families connected to the care team; and (3) care delivery services that include a full suite of escalation of care services, such as nurse practitioners, community paramedics, skilled nursing services, infusion therapy, phlebotomists, basic radiography technicians, and oxygen, dispatched to patients in their homes to allow for the provision of urgent and routine patient care needs.

Once a patient is randomized to the ACH arm, an admission huddle will be coordinated by the ACH physician, ACH RN, and service coordinator to plan patient-specific care and address care coordination needs. The patient will be physically transported home by community paramedics who set up the necessary technology and orient the patient and caregivers to that technology.

During the acute phase, daily rounds will be conducted by video with the ACH physician or in the patient’s home by APP with or without the presence of ACH physician by video. The physician and APPs huddle each day prior to rounds to determine if a patient is best served by an in-person visit, video visit, or both types of provider visits on the same day. Basic radiographic exams, such as x-rays and ultrasound, will be performed at home. Labs will be obtained and intravenous medications will be administered by a visiting nurse or community paramedic. Any advanced imaging or procedures will be accommodated by bringing the patient to the brick-and-mortar hospital and returning home upon completion. Any escalation of care to the hospital will be arranged as a transfer through the hospital admissions and transfer center. The endpoints for the acute phase are similar to the traditional hospital endpoints for discharge.

The next phase of care will be the restorative phase, which involves patient and family education, medication adherence, advanced care planning, and physical and occupational therapy to address patient-specific goals of care, such as optimizing medical and non-medical issues that result in hospitalization and monitoring for early signs of clinical decompensation. APPs will perform as-needed video and in-home visits and coordinate care during this phase of care. During the restorative phase, medications will be prescribed and sent to the patient’s preferred pharmacy. Any recommended outpatient clinical appointments will be coordinated to optimize their medical conditions. Near the end of the restorative phase, the discharge will be coordinated by the APPs and a handoff will be provided to the patient’s primary care physician. Home technology and equipment will be then removed, and the patient will be given discharge instructions prepared by the clinicians.

Of note, minor modifications to the care process or procedures are common in routine practice and could happen during the trial in both the ACH arm and the inpatient hospital care arm, due to quality improvement efforts or other operational needs. The project aims to compare two care models already in practice, and the research team does not interfere with or control clinical teams’ day-to-day operation. Local clinical and operational teams are responsible for communicating such modifications with patients and providers, but such changes in routine practice do not need to be reported to IRB. The principal investigators will work with the project manager and study coordinators to report to IRB regarding protocol deviation and other unexpected protocol amendments beyond routine practice changes and communicate with other relevant parties.

Due to the nature of the intervention (i.e., the delivery of acute care at home versus in the brick-and-mortar hospital), criteria for discontinuing or modifying allocated intervention are not needed. If patients in the ACH arm are transferred back to the hospital during the acute phase, such events will be recorded and reported as adverse events, and if patients are re-admitted during the restorative phase, such events will be counted in the outcome, i.e., 30-day readmission. A highly pragmatic trial would allow full flexibility in how the end-users engage with the intervention in order to estimate real-world effectiveness. Therefore, this trial will not utilize additional strategies to improve adherence above what are used in routine practice to engage patients. According to previous experience in practice, the cross-over between the two care delivery models has been rare. Any cross-over events will be recorded.

### Baseline measurements

Patient-level baseline data will be collected from the Mayo Clinic electronic health record (EHR), including age, sex, race, ethnicity, address, and the primary diagnosis for admission. These data will be ascertained at the time of randomization.

### Outcomes and follow-up

Follow-up will start from the day of randomization. It will contain two phases: the acute care phase (for the traditional inpatient care, this will last until the discharge from the hospital) and 30 days after the acute care phase. The primary outcome is a composite of all-cause mortality and 30-day readmission. The 30-day readmission will be assessed during the 30 days after the end of the acute phase. Transfers of ACH patients back to the hospital during the acute care phase will be recorded.

**Fig. 1:**
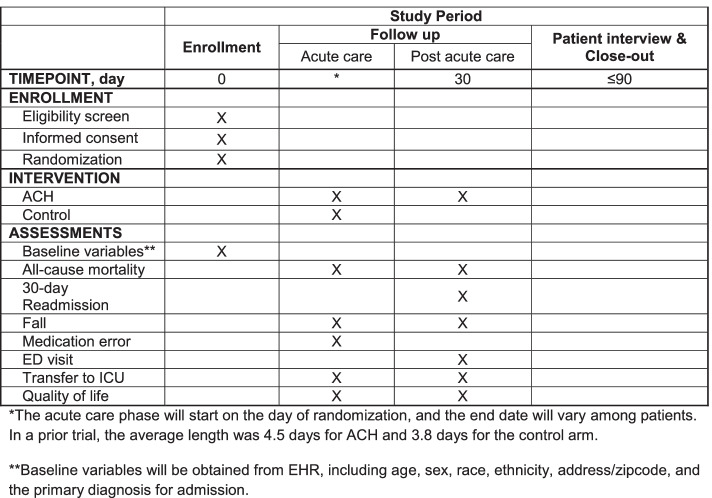
Schedule of enrollment, interventions, and assessments

Secondary outcomes include individual outcomes in the composite endpoint, fall with injury, medication error, emergency room visit, transfer to ICU, cost, the number of days alive out of the hospital, and quality of life. Fall with injury will be defined based on National Database for Nursing Quality Indicators criteria (Table 5). Medication error is defined in Table 6. Quality of life will be measured by the EQ-5D during the follow-up phone call (Fig. 1).

**Table 5:** Definition of fall with injury

Minor	Resulted in application of ice or dressing, cleaning of a wound, limb elevation, topical medication, pain, bruise, or abrasion
**Moderate**	Resulted in suturing, application of steri-strips or skin glue, splinting, or muscle/joint strain
**Major**	Resulted in surgery, casting, traction, required consultation for neurological (e.g., basilar skull fracture, small subdural hematoma) or internal injury (e.g., rib fracture, small liver laceration), or patients with any type of fracture regardless of treatment, or patients who have coagulopathy who receive blood products as a result of a fall
**Death**	The patient died as a result of injuries sustained from the fall (not from physiologic events causing the fall)

Fall with injury will be defined based on National Database for Nursing Quality Indicators (NDNQI) criteria. The primary analysis will consider all fall with injury and sensitivity analyses will be conducted to assess the severity of injury. All the clinical outcomes (e.g., fall and medication errors) are measured in routine practice. The research team will contact the clinical team if there is uncertainty about outcome adjudication

**Table 6:** Definition of medication error

Category F	An error occurred that may have contributed to or resulted in temporary harm to the patient and required initial or prolonged hospitalization
Category G	An error occurred that may have contributed to or resulted in permanent patient harm
Category H	An error occurred that required intervention necessary to sustain life
Category I	An error occurred that may have contributed to or resulted in the patient’s death.

The outcomes will be collected from the EHR, supplemented by a follow-up call from a study coordinator at the end of the study, i.e., after the 30th post-acute day. During this call, the study coordinator will ask the patient or the caregiver to ascertain outcomes, including mortality, readmission, fall with injury within 30 days, and medication errors. This follow-up call is necessary as some patients might receive care outside of Mayo Clinic not reflected in our EHR. If a participant has received care outside of Mayo Clinic, the study coordinator will send the participant an authorization for the release of records to sign and return by mail. The research team will contact relevant clinicians for additional outcome adjudication if needed. The study coordinator will also administer a 5-question measure of the quality of care (EQ-5D; Appendix EQ-5D) via phone [13], and a short survey to examine their care experience (Appendix Patient Care experience Survey) [14]. Patients and caregivers will receive up to three phone calls if they cannot be reached.

Exploratory studies will be conducted using EHR data to examine the same outcomes over longer periods of time, such as 91 days, 183 days, and one year.

### Patient experience and implementation

Semi-structured interviews will be conducted with a sample of patients/caregivers and clinicians/staff. We will use a purposeful sampling strategy to identify interviewees that represent each of the 3 sites and differing diagnosis groups. The number of patients and hospital staff is not pre-determined since qualitative research is concerned with the richness of the information rather than hypothesis testing. The data will be iteratively collected and analyzed; additional participants will be recruited as needed to understand emerging topics and explore variation. We expect to interview about 25–30 patients and 15–20 clinicians/staff. Patients will be recruited by phone after they have completed the study. Care team members will be invited via email to participate in an individual or group interview.

We will use the Systems Engineering Initiative for Patient Safety model [15] and the Consolidated Framework for Implementation Research [16, 17] to inform the qualitative data collection and analysis. A patient interview guide that was developed based on a previous study [18], focused on understanding patients’ experience with home hospital care (Additional file 6: Patient Interview Guide). A clinician and staff interview guide was developed to assess care team perceptions of ACH and to identify factors influencing implementation (Additional file 7: Clinician and Staff Interview Guide).

Interviews will last 45–60 min and be conducted by an experienced qualitative interviewer via telephone or video (depending on participant preference) and will be recorded. The recordings will be transcribed verbatim and analyzed using a content analysis approach [19, 20]. Two study members will review each interview transcript and make notes of their initial impressions (analytic memos). Those notes will be used to generate labels or codes, which will be arranged into higher-order categories related to the study aims. Preliminary analysis of the data will be concurrent with data collection to refine interview procedures to respond to emerging topics, and as part of our strategy to rapidly analyze data for timely dissemination and to inform intervention adaptations. Rapid analysis will leverage structured templates—organized around higher-order categories—to facilitate rapid synthesis of findings [21, 22]. Interviews will be examined within each subgroup as well as compared across groups (e.g., ACH vs. inpatient care), and the findings will be qualitatively described.

### Sample size

This randomized non-inferiority trial is designed to assess the non-inferiority of the defined ACH model compared to traditional inpatient hospital care. Based on preliminary analyses, we expect that the event proportion of the composite endpoint in the traditional brick-and-mortar hospital care arm will be approximately 30%. Additionally, we expect that the event rate in the ACH arm under the alternative hypothesis will be 30%. Sample size is based on the one-sided score test statistic for the non-inferiority test for an odds ratio of proportions. Given a non-inferiority odds ratio of 1.909, one-sided alpha of 0.025, and an interim analysis (defined under Interim Analysis), a sample size of 360 (180 per arm) is required to achieve 80% power. A non-inferiority margin expressed as an odds ratio of 1.909 with a 30% event rate in the traditional inpatient care arm is equivalent to a relative risk ratio of 1.5, a 45% event rate in the ACH arm, as the non-inferiority margin. EAST (Cytel) software was used for all sample size calculations.

### Statistical analysis

Logistic regression will be performed to assess the primary outcome. Analysis will be performed on all eligible patients who provide consent, are randomized, and start the intervention, following the intention-to-treat (ITT) principle. Cox proportional hazards regression analysis will also be performed to assess time-to-event; cumulative incidence curves will be plotted. If there is cross-over between treatment arms, a “per-protocol” analysis will be performed. In this per-protocol analysis, the ACH group would consider all patients who are randomized to ACH, with the follow-up censored at the time of crossing over to the control group; similarly, the control group would consider all patients who are randomized to control, with the follow-up censored at the time of crossing over to ACH. When analyzing continuous outcomes (i.e., cost and days alive out of hospital), a generalized linear model will be used, assuming a γ distribution with a log link, given the skewed nature of the data. When examining the long-term time-to-event outcomes beyond 30 days, a Cox proportional hazards model will be used when it may be necessary to account for censored data.

Because the baseline and outcome data will be obtained from the EHR, no significant missing data is expected. The study coordinators will check with patients during the end-of-study phone call regarding outcome events in case patients receive care outside of the study sites (Additional file 8). However, such events are anticipated to be rare in this patient population. In a previous trial of a HaH program, only 2 out of 91 patients received care outside the study sites [3].

Exploratory analyses such as machine learning approaches using causal tree will be performed to examine the heterogeneous treatment effect (HTE), i.e., which patients might benefit most from the intervention. Exploratory analyses will be performed stratified by acute and restorative phases.

### Interim analysis

An interim analysis will be performed when at least 50% of patients complete follow-up. We plan for this interim analysis to consider cost, resources, and meaningfulness of the trial and the interim analysis could call for potential termination or early declaration of success. Using the same composite endpoint and analysis plan as the primary endpoint, if the odds ratio of event proportions between the two arms is > 1.984, then the study will be stopped because one arm seems to be more beneficial than the other. Otherwise, the study will continue to full accrual and the final analyses will be conducted as described. The rho family spending function with rho=3.5 was used.

### Statistical analysis plan

The statistical analysis plan will be finalized before the close of the database. This plan will include all the analyses described above and other sensitivity and exploratory analyses. If there is any major deviation regarding the main analysis, an addendum to the protocol will be made. No changes will be made once the database is closed.

### Oversight and monitoring

The trial management group (TMG) includes XY, MSH, and EMB, which oversees the day-to-day operation of the trial and meets daily. The trial steering committee (TSC) includes XY, SMD, SCD, EBH, MP, MJM, AND, CRN, and LRS and is responsible for making executive decisions, providing advice, and endorsing the TMG actions. An independent DSMB has been assembled to act in an advisory capacity to monitor participant safety, data quality, and progress. The DSMB charter is provided in Additional file 9.

### Safety reporting

Due to the nature of the interventions, i.e., comparing two models of delivering acute care in routine practice, the anticipated adverse events are included as the primary and secondary outcomes and will be managed as part of the routine care. Any unanticipated adverse events should be reported to TMG as soon as possible within 24 h. All the adverse events, regardless of whether anticipated or not, will be reported to DSMB.

### Data management

Throughout the trial, the PI (XY), project manager (MSH), and protocol specialist/data manager (EMB) will monitor the quality of the trial with special attention to protocol deviations and the quality of data entered in the REDCap database. MSH will send the study sites and TSC an update of the trial progress every 2 weeks. Protocol deviations will be communicated with TSC, IRB, and study sites as needed. Certain functions have been built in REDCap to automatically prevent logical inconsistencies. EMB will perform manual data auditing within 45 days of the enrollment of the first patient and repeat the auditing at least quarterly and at the end of the study. During each audit, five patients will be randomly selected and checked for any previously unrecognized missingness, errors, or logical inconsistencies. An auditing log will be maintained to document dates of auditing, patient identifier, and any problems uncovered from the auditing. At the end of the trial after the final auditing is completed, a data management meeting will be held to assess the suitability of the database for analysis, and the database will be then closed.

## Discussion

The ACH virtual hybrid HaH model has great potential to improve patient outcomes and reduce costs, but few rigorous evaluations of this new care delivery model exist. The only recent RCT in the USA enrolled 91 patients in one urban setting, thereby lacking the ability to definitely ascertain the effectiveness and safety [3]. The lack of evidence may prohibit or delay providers and payers to adopt this new model of high-acuity home care, especially in suburban and rural areas. We will conduct a pragmatic trial to inform real-world decisions regarding alternative treatment options in the delivery of advanced health care to acutely ill adults.

**Fig. 2:**
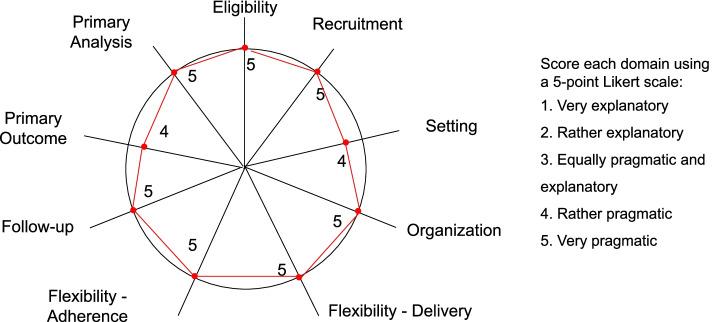
Evaluation of pragmatism—PRECIS-2 Wheel

Pragmatism is a continuum that can be assessed by the PRECIS-2 tool from nine domains, each of which is rated from 1 (very explanatory) to 5 (very pragmatic) [23]. The PRECIS-2 tool is encouraged to be used at the design stage to ensure that the design choices are concordant with the intended purpose. Therefore, we report the evaluation of pragmatism below (Fig. 2).

The first domain of pragmatism is “eligibility”—“To what extent are trial participants similar to those who would receive this intervention if it was part of usual care?” The ACH trial aims to enroll all patients who are at the equipoise of receiving ACH or traditional brick-and-mortar hospital care. The trial eligibility criteria aim to exclude patients who would likely only qualify for one arm, not both, and were determined by the clinical team, not the research team. Therefore, the trial is very pragmatic, with a score of 5, aiming to represent the population to which the results will apply.

The second domain is “recruitment”: “How much extra effort is made to recruit participants over and above what would be used in the usual care setting to engage patients?” A highly pragmatic approach would be to recruit through usual appointments at diverse clinics and hospitals—exactly what the ACH trial will do. Local clinical teams will screen all patients as they do in routine practice. Once an eligible patient is identified, the local clinical team will contact the central research team at Mayo Clinic, Rochester, Minnesota. If patients refuse to participate in the research study, then they will continue with their routine care of choice, either ACH or traditional brick-and-mortar hospital care. In other words, we will embed the recruitment into the routine clinical workflow, and the research activities (e.g., introducing the study, research consent, and randomization) will be conducted virtually by the central research team, thereby minimizing the burden on local clinical staff. As such, the trial is very pragmatic in terms of recruitment, with a score of 5.

The third domain is “setting”—“How different are the settings of the trial from the usual care setting?” We will perform the trial at three hospitals in Arizona, Florida, and Wisconsin, including two academic medical center hospitals and one community-based hospital. The trial setting mimics routine practice and is geographically diverse. However, all three hospitals belong to Mayo Clinic’s multistate health system. Therefore, the trial is rather pragmatic in terms of setting, with a score of 4.

The fourth domain is “organization”—“What expertise and resources are needed to deliver the intervention?” The ACH trial will make use of no more than the existing staff and resources in routine practice and, thus, is very pragmatic with a score of 5.

The fifth domain is “flexibility in terms of the delivery of the intervention.” The ACH trial will leave the details of how to deliver the intervention to frontline clinicians and staff, rather than being rigidly prescriptive in the protocol. Therefore, the trial is very pragmatic, with a score of 5.

The sixth dimension is “flexibility in terms of adherence.” ACH will allow full flexibility in how the end-users engage with the intervention. Therefore, it is very pragmatic with a score of 5.

The seventh dimension is follow-up. In the ACH trial, baseline characteristics will be captured from the EHR, and outcome data will be collected via a combination of EHR and a study coordinator call enabling patient reporting. We will conduct a one-time interview at the end of the study. This hybrid approach using both EHR and patient reporting is to improve the completeness of the data, e.g., mortality, readmission, and other events outside the study sites, as well as to collect patient-reported outcomes. Therefore, we consider the trial very pragmatic with a score of 5.

The eighth dimension is “primary outcome”—measures “How relevant is the outcome to participants?” The primary outcome is the composite of all-cause mortality and readmission. The outcome is clearly important for patients, clinicians, and our health system, but since it is a composite outcome, we will give a score of 4 as “rather pragmatic” for this domain.

The ninth dimension is primary analysis. The ACH trial will use the intention-to-treat analysis with all the available data as the primary analysis and, thus, is very pragmatic with a score of 5.

The current study is also an example of building a learning health system, which is characterized by continual improvement and innovation with new knowledge captured as an integral by-product of the delivery experience [24, 25]. A variety of HaH programs have been developed in other health systems over the past several decades. Mayo Clinic leveraged innovative technologies and care delivery methods to further improve this model. Traditionally, such new care delivery innovations and quality improvement programs would be implemented as is because they seem like good ideas, but few were rigorously evaluated. Recently, a few pragmatic trials demonstrated that the programs with face validity might not improve care delivery and patient outcomes when evaluated rigorously [26, 27]. Therefore, multiple institutions have transitioned towards a learning health system through rapid-cycle evaluation embedded into routine clinical care.

Such pragmatic approaches could generate evidence to inform real-world decision-making at a low cost and fast pace. However, unlike conventional clinical trials, these projects are mainly conducted by internal teams, due to the short turnaround time, cost, and resource constraint. Furthermore, most of the innovations might lead to business development opportunities. For example, a health system might hold patents for the artificial intelligence algorithms they developed, or in this trial, Mayo Clinic has financial investment in a company that supports the new care delivery model. As a result, how to avoid bias in evaluation needs to be considered. A common approach is for a health system to have a dedicated independent team serving as the “unbiased evaluator.” This evaluation team does not work exclusively in any particular disease area and has no financial interest or other relationships with the departments that developed the innovations. For example, NYU Langone Health’s team was able to complete ten randomized rapid cycle testing within a year [24]; similarly, the current trial is led by Mayo Clinic Kern Center for the Science of Health Care Delivery, which has evaluated a broad range of interventions using prospective and retrospective data, as well as quantitative and qualitative methodologies [28–30].

In conclusion, the current study will be the first large pragmatic RCT that examines the HaH model in diverse routine clinical practice settings. The pragmatism is high with all the domains having a score of at least 4. The results will inform whether the ACH model maintains or improves the quality and safety of patient care traditionally delivered at inpatient settings. The findings will also identify barriers and needs from patients, clinicians, and other staff, thereby paving the way for future large-scale implementation.

### Trial status

The trial is currently under way, with recruitment having commenced in February 15, 2022. Protocol version: 3 (December 5, 2021). Recruitment is likely to be completed in 2023.

## Supplementary Information

**Additional file 1.** SPIRIT checklist

**Additional file 2.** WHO Trial Registration Dataset

**Additional file 3.** Clinical Stability Screen

**Additional file 4.** Social Stability Screening

**Additional file 5.** Informed consent

**Additional file 6.** Patient Interview Guide

**Additional file 7.** Clinician and Staff Interview Guide

**Additional file 8.** Patient 30-Day Follow-Up

**Additional file 9.** ACH DSMB Charter

## Data Availability

The trial findings will be presented in conferences and published in peer-reviewed journals. The statistical codes will be published with the main trial results paper. SAI and EMB will have access to the final trial dataset. Request of individual-level dataset can be made directly to the contact author and reviewed on a case-by-case basis.

## Abbreviations

ACH: Advanced care at home
APP: Advanced practice provider
DSMB: Data and Safety Monitoring Board
ED: Emergency department
EHR: Electronic health record
HTE: Heterogeneous treatment effect
ICU: Intensive care unit
ITT: Intention to treat
IRB: Institutional Review Board
REDCap: Remote Data Capture
RCT: Randomized controlled trial
RN: Registered nurses
TMG: Trial management group
TSC: Trial steering committee
