# Supplementary material for: Effect of hospital-at-home vs. traditional brick-and-mortar hospital care in acutely ill adults: study protocol for a pragmatic randomized controlled trial
Source: Trials. 2022 Jun 16;23:503. doi: 10.1186/s13063-022-06430-6 (PMC9201794; doi:10.1186/s13063-022-06430-6)
Supplement: Supplementary file 2 — Additional file 2. WHO Trial Registration Dataset [file 13063_2022_6430_MOESM2_ESM.docx]

**Additional File 2. World Health Organization Trial Registration Data Set**

| **Data category** | **Information** |
| --- | --- |
| 1. Primary registry and trial identifying number | ClinicalTrials.gov |
| 1. Date of Registration in Primary Registry | NCT05212077 |
| 1. Secondary identifying numbers | 21-005335 |
| 1. Source(s) of Monetary or Material Support | Mayo Clinic |
| 1. Primary Sponsor | Mayo Clinic |
| 1. Secondary Sponsor(s) | None |
| 1. Contact for Public Queries | Emma M. Behnken behnken.emma@mayo.edu |
| 1. Contact for Scientific Queries | Xiaoxi Yao yao.xiaoxi@mayo.edu |
| 1. Public Title | Providing hospital-level care at home in acutely ill patients |
| 1. Scientific Title | Effect of Advanced Care at Home vs. Traditional Brick-and-Mortar Hospital Care in Acutely Ill Adults: a pragmatic randomized controlled trial |
| 1. Countries of Recruitment | United States |
| 1. Health Condition(s) or Problem(s) Studied | To compare two acute care delivery models |
| 1. Intervention(s) | a novel “Hospital at Home” program, i.e., Advanced Care at Home (ACH), which combines a virtual physician-staffed command center with a vendor-mediated supply chain that can deliver high-acuity care |
| 1. Key Inclusion and Exclusion Criteria | The eligibility criteria will follow what would be used in routine practice at the time of enrollment. The frontline clinicians will make the decision, i.e., identifying patients at clinical equipoise who can benefit from either ACH or traditional inpatient care, and will refer eligible patients to the study coordinator for consent and randomization.  Inclusion Criteria:   - Adult patients, 18 years of age and older. - Present to one of the participating hospitals. - Have a chief complaint of one of the target diagnoses. - Are within a certain geographical area (based on zip codes). - Have a health insurance plan that covers ACH services. - Have the capacity to consent or could assent with the consent of a health care proxy who is physically present.   Exclusion Criteria:   - The patient is not suitable for ACH or inpatient hospital care based on: - being a nursing home patient; - on or requiring dialysis; - positive for COVID-19; - having discharge order; - requiring intensive care unit (ICU) level of care; - history of drug abuse. - Patients will also need to meet the clinical stability criteria. - Do not have the capacity to consent or assent with the assistance of a health care proxy. |
| 1. Study Type | Interventional  Allocation: Randomized; Parallel Assignment  Masking: None (Open Label) |
| 1. Date of First Enrollment | February 15, 2022 |
| 1. Target Sample Size | 360 |
| 1. Recruitment Status | Recruiting |
| 1. Primary Outcome(s) | A composite outcome of all-cause mortality and 30-day readmission (time frame: acute phase and 30 days after the end of the acute phase) |
| 1. Key Secondary Outcomes | Individual outcomes in the composite endpoint, fall with injury, medication errors, emergency room visit, transfer to intensive care unit (ICU), cost, the number of days alive out of hospital, and patient-reported quality of life |
| 1. Ethics Review | Approved by Mayo Institutional Review Board (IRB) January 18, 2022 |
