# Supplementary material for: Effect of hospital-at-home vs. traditional brick-and-mortar hospital care in acutely ill adults: study protocol for a pragmatic randomized controlled trial
Source: Trials. 2022 Jun 16;23:503. doi: 10.1186/s13063-022-06430-6 (PMC9201794; doi:10.1186/s13063-022-06430-6)
Supplement: Supplementary file 3 — Additional file 3. Clinical Stability Screen [file 13063_2022_6430_MOESM3_ESM.pdf]

Medically Home

Clinical Stability Screen

Must be NO:	Yes	No	Comment
Low diagnostic certainty requiring advanced diagnostics that cannot be performed in the home<br><br>If yes, select reason (if "other", select and describe):<br><br>☐ Active live telemetry monitoring<br>☐ Advanced imaging required (CT, MRI, nuclear stress)<br>☐ Cardiac catheterization required<br>☐ EGD/Colonoscopy required<br>☐ Lab monitoring not amenable to home (troponin, >q12 labs)<br>☐<br>Other____			
Higher acuity medical services may be required making admission to home clinically unsafe<br><br>If yes, select reason (if "other", select and describe):<br><br>☐ Intensive care unit required (i.e. vitals checked >q4hr, BG checked >q2hr, active drip titration etc.)<br>☐ Intubation risk elevated (i.e. history of intubation for respiratory conditions)<br>☐ New tracheostomy or mechanical ventilation required<br>☐ Vasopressor risk elevated (i.e. reasonable risk of fluid refractory hypotension)<br>☐ Unstable arrhythmias<br>☐ Elevated risk of respiratory compromise increasing risk of needing support with noninvasive positive pressure ventilation or intubation<br>☐ Home IV access limitations<br>☐<br>Other____			
Specific services required that cannot be Provided in the Home<br><br>If yes, select reason (if "other", select and describe):<br><br>☐ Risk for inability to obtain IV access<br>☐ Daily involvement of in person specialty consultation<br>☐ Blood transfusions likely to be needed (i.e. active GI bleed)<br>☐ Hemodialysis likely to be required (i.e. already on HD or newly anuric/severe ATN)			

☐ Respiratory isolation/airborne precautions (high TB concern)<br>☐ Pain such that would require adjustment of opiates or need for IV therapy<br>☐ Chronic need for 24/7 care that is currently not in place in the home setting<br>☐<br>Other____			
Surgical/IR procedure required that cannot be performed in the home<br><br>If yes, select reason (if "other", select and describe):<br><br>☐ Surgical consultation required or high risk of being required<br>☐ Intraabdominal abscess requiring drainage (an example)<br>☐ Percutaneous nephrostomy tube placement (an example)<br>☐ Thoracentesis for parapneumonic effusion (an example)<br>☐ Significant surgical wound debridement<br>☐<br>Other____<br>__			
Clinical partnership with patient and family untenable to safely care for patient<br><br>If yes, select reason (if "other", select and describe):<br><br>☐ Severe altered mental status unable to be managed safely at home with family and home health aide (an example)<br>☐ Active substance abuse (an example)<br>☐ History of or high risk of noncompliance with primary treatment plan<br>☐<br>Other____<br>__			
Is the patient currently on hospice or have a life expectancy of <= 6 mo.?			
