# Supplementary material for: Effect of hospital-at-home vs. traditional brick-and-mortar hospital care in acutely ill adults: study protocol for a pragmatic randomized controlled trial
Source: Trials. 2022 Jun 16;23:503. doi: 10.1186/s13063-022-06430-6 (PMC9201794; doi:10.1186/s13063-022-06430-6)
Supplement: Supplementary file 4 — Additional file 4. Social Stability Screening [file 13063_2022_6430_MOESM4_ESM.pdf]

Medically Home

Social Stability Screen

Complete for ALL Programs

**Must be YES:**	**Yes**	**No**	**N/A**	**Comment**
Does the patient have a home recovery environment?				
Is the patient's home recovery environment in an eligible geography?				
Does the patient's home have water, electricity, bathroom, heat/ac, refrigerator?				
Does the patient feel safe at home?				
Are family/caregivers willing to participate, as needed, while the patient participates in the MH program?				
Is there a person in the home (patient or other) willing/able to take vital signs and answer phone calls?				
Is the patient willing to put pets in a secure area while MH affiliated staff are in the home?				
Is patient willing/able to participate in the MH program (this includes MH affiliated staff entering the home, and MH home equipment set-up in the home)?				
Is the patient/patient's HCP willing/able to sign consent?				

**Informative Questions:**	**Yes**	**No**	**N/A**	**Comment**
Does the patient have internet at home and a cell phone?				
Does the patient live in a stand-alone house or in a community setting? *Options: house, apartment, other*				
What floor does the patient live on?				
How many steps are there to enter the home?				
Is there an elevator at the home?				
Are there stairs within the home? *If yes-how many?*				
Does the patient live alone?				
Is there anyone who assists the patient with day to day life? *If yes-describe*				
Does the patient currently have any home services?				
Is the patient likely to require >4 hrs. of HHA support to fill ADL needs?				
Does the patient have mild, or greater, cognitive impairment?				
Has the patient experienced a fall within the last 6 mo.? *If yes, explain:*				
Is the patient homebound?				
Is the NIDA Quick Screen positive for the patient? *(see below for NIDA Quick Screen; if positive- explain)*				

Is the NIDA Quick Screen positive for the anyone living in the patient's home? *(see below for NIDA Quick Screen; if positive- explain)*				
Is the patient's primary language English? *If no, what is the patient's primary language?*				

**Must be NO:**	**Yes**	**No**	**N/A**	**Comment**
Are there any behavioral diagnosis that would limit patient participation in the MH program?				
Does the patient require 1:1 aid with feeding or daily speech therapy?				
Does the patient require 1:1 aid with toileting and lack home assistance?				
Is the patient or caregiver unable to call for help or push the emergency help device?				
Is there active smoking in the home and need for home Oxygen?				
Are there unsecured firearms in the home?				
Does the patient exhibit signs of neglect suggestive of an unsafe home?				
Is the patient deaf?				

NIDA Quick Screen:

In the past year, how many times have you used the following….

	**Never**	**Once or Twice**	**Monthly**	**Weekly**	**Almost Daily or Daily**	**Comment**
Alcohol (for men, 5 or more drinks in a day; for women, 4 or more drinks in a day)						
Tobacco Products						
Prescription Drugs for Nonmedical Reasons						
Illegal Drugs (if in a state where marijuana is legal, specifically ask re: marijuana use)						

<u>Complete for SNF Substitution Program ONLY</u>

**Must be YES:**	**Yes**	**No**	**N/A**	**Comment**
Has the patient been recommended for discharge to SNF?				

Are the patient/caregivers aware that HHA support provided by the MH program is temporary?				
Does the patient's home have sufficient space/appropriate condition for rehab needs?				
Are caregivers willing to aid the patient w/ADLs during the patient's time in the MH program and post d/c from the MH program (if needed)?				

**Must be NO:**	**Yes**	**No**	**N/A**	**Comment**
Has the patient been previously recommended for long-term care?				
Does the patient live at a SNF/SAR/LTAC?				
Does the patient no longer prefer to live at home after the hospital/SNF stay?				
Has the patient required a 1:1 sitter in the last 48 hours?				
Has PT deemed the patient to be either a maximum assist or two assists in any functional category?				

Complete for ALL Programs

**Must be YES:**	**Yes**	**No**	**N/A**	**Comment**
Did the patient pass this screen?				
