# Supplementary material for: Effect of hospital-at-home vs. traditional brick-and-mortar hospital care in acutely ill adults: study protocol for a pragmatic randomized controlled trial
Source: Trials. 2022 Jun 16;23:503. doi: 10.1186/s13063-022-06430-6 (PMC9201794; doi:10.1186/s13063-022-06430-6)
Supplement: Supplementary file 5 — Additional file 5. Informed consent [file 13063_2022_6430_MOESM5_ESM.pdf]

MAYO CLINIC

Approval Date: **January 14, 2022**
Not to be used after: **January 13, 2023**

**Name and Clinic Number**

RESEARCH PARTICIPANT CONSENT AND PRIVACY AUTHORIZATION FORM

Study Title: Effect of Advanced Care at Home vs. Traditional Brick-and-Mortar Hospital Care in Acutely Ill Adults: A Randomized Clinical Trial

IRB#: 21-005335

Principal Investigator: Xiaoxi Yao, Ph.D. and Colleagues

Key Study Information

This section provides a brief summary of the study. It is important for you to understand why the research is being done and what it will involve before you decide. **Please take the time to read the entire consent form carefully and talk to a member of the research team before making your decision.** You should not sign this form if you have any questions that have not been answered.	
**It's Your Choice**	This is a research study. Being in this research study is your choice; you do not have to participate. If you decide to join, you can still stop at any time. You should only participate if you want to do so. You will not lose any services, benefits or rights you would normally have if you choose not to take part.
**Research Purpose**	The purpose of this research is to better understand and compare two kinds of hospitalization that are currently being implemented in clinical practice.<br><br>You are being invited to participate in this study because you are being considered for hospitalization for an acute illness.
**What's Involved**	Study participation involves being assigned by chance (like a coin toss) to the traditional inpatient hospitalization group or the Advanced Care at Home (ACH) group. You will have access to the same services in either group. Services will be offered in the hospital setting for participants randomized to inpatient hospitalization, and services will be offered in the home for participants randomized to the Advanced Care at Home (ACH) model of care.

	Name and Clinic Number

Approval Date: **January 14, 2022**
Not to be used after: **January 13, 2023**

**Key Information**	The risks in this study may vary depending on which group you are assigned to, and we do not believe they will be any different than what you would experience as part of your routine clinical care. Your care team will discuss the risks of any tests/procedures performed, as any tests and procedures are being done as part of your clinical care.<br><br>This study may not make your health better. However, future patients may benefit from what we learn in this study.<br><br>You don't have to be in this study to enroll in the ACH program or continue with traditional inpatient hospitalization for your condition.
**Learn More**	If you are interested in learning more about this study, read the rest of this form carefully. The information in this form will help you decide if you want to participate in this research or not. A member of our research team will talk with you about taking part in this study before you sign this form. If you have questions at any time, please ask us.

Making Your Decision

Taking part in research is your decision. Take your time to decide. Feel free to discuss the study with your family, friends, and healthcare provider before you make your decision. Taking part in this study is completely voluntary and you do not have to participate.

If you decide to take part in this research study, you will sign this consent form to show that you want to take part. We will give you either a printed or electronic copy of this form to keep.

If you are signing this consent form for someone else, "you" in the consent form refers to the participant.

For purposes of this form, Mayo Clinic refers to Mayo Clinic in Arizona, Florida and Rochester, Minnesota; Mayo Clinic Health System; and all owned and affiliated clinics, hospitals, and entities.

MAYO CLINIC

Approval Date: **January 14, 2022**
Not to be used after: **January 13, 2023**

Name and Clinic Number

Contact Information

If you have questions about …	You can contact …
▪ Study tests and procedures<br>▪ Materials you receive<br>▪ Research-related appointments<br>▪ Research-related concern or complaint<br>▪ Research-related injuries or emergencies<br>▪ Withdrawing from the research study	**Principal Investigator(s):** Xiaoxi Yao, Ph.D.<br>**Phone:** (507) 266-5434<br><br>**Study Team Contact:** Emma Behnken<br>**Phone:** (507) 293-0177<br><br>**Institution Name and Address:**<br>Mayo Clinic<br>200 First Street SW<br>Rochester, MN 55905
▪ Rights of a research participant	**Mayo Clinic Institutional Review Board (IRB)**<br>**Phone:** (507) 266-4000<br>**Toll-Free:** (866) 273-4681
▪ Rights of a research participant<br>▪ Any research-related concern or complaint<br>▪ Use of your Protected Health Information<br>▪ Stopping your authorization to use your Protected Health Information<br>▪ Withdrawing from the research study	**Research Subject Advocate (RSA)**<br>**(The RSA is independent of the Study Team)**<br>**Phone:** (507) 266-9372<br>**Toll-Free:** (866) 273-4681<br><br>**E-mail:** researchsubjectadvocate@mayo.edu
▪ Billing or insurance related to this research study	**Patient Account Services**<br>**Toll-Free:** (844) 217-9591

Other Information:

A description of this clinical trial will be available on http://www.ClinicalTrials.gov, as required by U.S. Law. This Web site will not include information that can identify you. At most, the Web site will include a summary of the results. You can search this Web site at any time.

Why are you being asked to take part in this research study?

You are being invited to participate in this study because you are being considered for hospitalization for an acute illness.

Why is this research study being done?

This research is being done to better understand and compare two kinds of hospitalization that are currently being implemented in clinical practice.

Information you should know

Who is Funding the Study?

The Mayo Clinic Robert D. and Patricia E. Kern Center for the Science of Health Care Delivery are providing the funding for this study.

Information Regarding Conflict of Interest:

If your healthcare provider is also an investigator on this study, there is the chance that his or her responsibilities for the study could influence his or her recommendation for your participation. If you prefer, your healthcare provider will be happy to refer you to another investigator on the research study team for you to decide if you want to participate in the study and to see you for the research study activities while you are in the study.

How long will you be in this research study?

Your active participation in this study will last for about one month.

What will happen to you while you are in this research study?

If you agree to be in the study, we will assign you by chance (like a coin toss) to the Advanced Care at Home (ACH) group or the traditional inpatient hospitalization group. You and the Principal Investigator can't choose your study group. You will have an equal chance of being assigned to the ACH group.

Participants in both groups will receive access to the same services. Services will be offered in the hospital setting for participants randomized to traditional inpatient hospitalization, and services will be offered in the home for participants randomized to the Advanced Care at Home (ACH) model of care.

If you are randomized to enroll in the Advanced Care at Home (ACH) model of care, your care may include in-home visits by a nurse practitioner or physician assistant and/or other health care professionals (as determined by your care team). You will be provided with a computer tablet for video visits with your Mayo Clinic are team, a telephone that connects directly to your care team, a personal emergency response bracelet, virtual sign monitoring devices and a backup power supply. Lab tests, mobile imaging like ultrasounds and x-rays, and IV therapies can also be performed in your home. During care, just as if you were in the hospital, we ask that you stay home. Your individual diagnosis and circumstances will be taken into consideration by the care team should you need to leave your home during your care. When you are well enough to be discharged, your Mayo Clinic care team will ensure a smooth transition out of the program and the removal of all the home care technology.

Participants in both groups will receive a follow-up phone call from a study coordinator around month into participation. At the time of this phone call, the study coordinator will ask questions regarding the care you received during hospitalization.

You may also be asked to participate in an interview with a member of our research team to discuss your hospitalization experience. This interview will last for about one hour, and it will take place at a time that is convenient for you. The interview discussed will be audio-recorded and transcribed.

What are the possible risks or discomforts from being in this research study?

The risks in this study may vary depending on which group you are assigned to, and we do not believe they will be any different than what you would experience as part of your routine clinical care.

Your care team will discuss the risks of any tests/procedures performed, as any tests and procedures are being done as part of your clinical care.

As with all research, there is a chance that confidentiality could be compromised; however, we take precautions to minimize this risk.

Are there reasons you might leave this research study early?

You may decide to stop at any time. You should tell the Principal Investigator if you decide to stop and you will be advised whether any additional tests may need to be done for your safety.

In addition, the Principal Investigator or Mayo Clinic may stop you from taking part in this study at any time:

- If it is in your best interest,
- If you don't follow the study procedures,
- If the study is stopped.

If you leave this research study early, or are withdrawn from the study, no more information about you will be collected; however, information already collected about you in the study may continue to be used.

We will tell you about any new information that may affect your willingness to stay in the research study.

What if you are injured from your participation in this research study?

Where to get help:

If you think you have suffered a research-related injury, you should promptly notify the Principal Investigator listed in the Contact Information at the beginning of this form. Mayo Clinic will offer care for research-related injuries, including first aid, emergency treatment and follow-up care as needed.

Who will pay for the treatment of research related injuries?

Care for such research-related injuries will be billed in the ordinary manner, to you or your insurance. You will be responsible for all treatment costs not covered by your insurance, including deductibles, co-payments and coinsurance.

What are the possible benefits from being in this research study?

This study may not make your health better. However, future patients may benefit from what we learn in this study.

What alternative do you have if you choose not to participate in this research study?

You don’t have to be in this study to enroll in the ACH program or continue with traditional inpatient hospitalization for your condition. Talk to the Principal Investigator or your doctor if you have any questions about any of these treatments or procedures.

What tests or procedures will you need to pay for if you take part in this research study?

You and/or your insurance will need to pay for all tests and procedures that you would have as part of your clinical care, including co-payments and deductibles.

If you have billing or insurance questions call Patient Account Services at the telephone number provided in the Contact Information section of this form.

Will you be paid for taking part in this research study?

You won't be paid for taking part in this study.

Will your information or samples be used for future research?

Unless you give your permission below, your information or samples collected for this study will not be used or shared for future research, even if the identifiable information such as your name, Mayo Clinic number or date of birth is removed.

We would like to keep your information for future research. You can still take part in this current study even if you don't want your information used for future research.

Researchers at Mayo Clinic who aren't involved with this study may ask to use your information for future research. Researchers at other institutions may also ask for a part of your information for future studies. Unless you indicate otherwise, the future research may be on any topic. No direct benefits to you are expected from the future research. Your information will only be shared consistent with your consent, and with all applicable laws and regulations.

Name and Clinic Number

If you approve release of your information by checking ‘yes’ below, Mayo may send the information to researchers who request them, but Mayo will not send your name, address, phone number, social security number, or any other identifying information with the information. Your information may be sent with a code, and only the researchers for this study at Mayo Clinic would be able to link the code to you.

Please read the following statements and mark your choices:

1. I permit my information to be stored and used in future research at Mayo Clinic to learn about, prevent, or treat any other health problems:

☐ Yes ☐ No Please initial here: ________Date: ________

2. I permit Mayo Clinic to give my information to researchers at other institutions:

☐ Yes ☐ No Please initial here: ________Date: ________

You may withdraw your consent for future use of your information and at any time, by writing to the Principal Investigator at the address provided in the “Contact Information" section of this consent form.

Your information would be removed from any repository where they are stored, if possible. Information already distributed for research use will not be retrieved.

How will your privacy and the confidentiality of your records be protected?

Mayo Clinic is committed to protecting the confidentiality of information obtained about you in connection with this research study.

Your privacy is very important to us. We follow several procedures in order to protect your confidentiality. The electronic files in this study will be immediately transferred to secure and password-protected servers which only authorized research personnel will have access. Similarly, any paper files will be stored in locked file cabinets to which only select personnel have access.

During this research, information about your health will be collected. Under Federal law called the Privacy Rule, health information is private. However, there are exceptions to this rule, and you should know who may be able to see, use, and share your health information for research and why they may need to do so. Information about you and your health cannot be used in this research study without your written permission. If you sign this form, it will provide that permission (or "authorization") to Mayo Clinic.

Your health information may be collected from:

- Past, present and future medical records.
- Research procedures, including the monitoring test, and questionnaires.

Your health information will be used and/or given to others to:

- Do the research.
- Report the results.
- See if the research was conducted following the approved study plan, and applicable rules and regulations.

Your health information may be used and shared with:

- Mayo Clinic research staff involved in this study.
- Other Mayo Clinic staff involved in your clinical care.
- The sponsor of this study (i.e. Mayo Clinic) and the people or groups hired by the sponsor(s) to help perform this research.
- The Mayo Clinic Institutional Review Board that oversees the research.
- Federal and State agencies (such as the Food and Drug Administration, the Department of Health and Human Services, the National Institutes of Health and other United States agencies) or government agencies in other countries that oversee or review research.
- A group that oversees the data (study information) and safety of this research.

How your information may be shared with others:

While taking part in this study, you will be assigned a code that is unique to you, but does not include information that directly identifies you. This code will be used if your study information is sent outside of Mayo Clinic. The groups or individuals who receive your coded information will use it only for the purposes described in this consent form.

If the results of this study are made public (for example, through scientific meetings, reports or media), information that identifies you will not be used.

In addition, individuals involved in study oversight and not employed by Mayo Clinic may be allowed to review your health information included in past, present, and future medical and/or research records. This review may be done on-site at Mayo Clinic or remotely (from an off-site location). These records contain information that directly identifies you. However, the individuals will not be allowed to record, print, or copy (using paper, digital, photographic or other methods), or remove your identifying information from Mayo Clinic.

Is your health information protected after it has been shared with others?

Mayo Clinic asks anyone who receives your health information from us to protect your privacy; however, once your information is shared outside Mayo Clinic, we cannot promise that it will remain private and it may no longer be protected by the Privacy Rule.

Your Rights and Permissions

Participation in this study is completely voluntary. You have the right not to participate at all. Even if you decide to be part of the study now, you may change your mind and stop at any time. You do not have to sign this form, but if you do not, you cannot take part in this research study.

Deciding not to participate or choosing to leave the study will not result in any penalty. Saying 'no' will not harm your relationship with your own doctors or with Mayo Clinic.

If you cancel your permission for Mayo Clinic to use or share your health information, your participation in this study will end and no more information about you will be collected; however, information already collected about you in the study may continue to be used.

You can cancel your permission for Mayo Clinic to use or share your health information at any time by sending a letter to the address below:

Mayo Clinic
Office for Human Research Protection
ATTN: Notice of Revocation of Authorization
201 Building 4-60
200 1st Street SW
Rochester, MN 55905

Name and Clinic Number

Approval Date: January 14, 2022
Not to be used after: January 13, 2023

Alternatively, you may cancel your permission by emailing the Mayo Clinic Research Subject Advocate at: researchsubjectadvocate@mayo.edu.

Please be sure to include in your letter or email:

- The name of the Principal Investigator,
- The study IRB number and /or study name, and
- Your contact information.

Your permission for Mayo Clinic to use and share your health information lasts until the end of this study, unless you cancel it. The study does not end until all data has been collected, checked (or audited), analyzed, and reported. Because research is an ongoing process, we cannot give you an exact date when the study will end. Sometimes this can be years after your study visits and/or activities have ended.

Enrollment and Permission Signatures

Your signature documents your permission to take part in this research.

	/ /	: AM/PM
Printed Name	Date	Time

____
Signature

Signature of Legally Authorized Representative for Adult Participant

- I give permission for the participant to take part in this research study and agree to allow his/her health information to be used and shared as described above.

				AM/PM
Signature	Printed Name	Relationship to Participant	Date	Time

MAYO CLINIC

Approval Date: January 14, 2022
Not to be used after: January 13, 2023

Name and Clinic Number

Person Obtaining Consent

- I have explained the research study to the participant.
- I have answered all questions about this research study to the best of my ability.

/ / : AM/PM

Printed Name Date Time

Signature

Signature of a witness to the consent conversation:

I observed the entire consent conversation and confirm that the participant appears to understand the information and was given the opportunity to ask questions.

/ / : AM/PM

Witness (Print Name) Date Time

Signature of Witness
