# Supplementary material for: Effect of hospital-at-home vs. traditional brick-and-mortar hospital care in acutely ill adults: study protocol for a pragmatic randomized controlled trial
Source: Trials. 2022 Jun 16;23:503. doi: 10.1186/s13063-022-06430-6 (PMC9201794; doi:10.1186/s13063-022-06430-6)
Supplement: Supplementary file 6 — Additional file 6. Patient Interview Guide [file 13063_2022_6430_MOESM6_ESM.docx]

**Patient Interview Guide**

Mr./Ms._____________, thank you for taking the time to talk with me and for agreeing to participate in this study.

My name is ________________, a ___________ at Mayo Clinic.

This study was a first step toward changing the way we take care of adults who become acutely ill. Having recently been admitted, you are truly a (home) hospital expert. We are hoping to gain insight into your (home) hospital experience so that we can improve it moving forward.

All of your responses will be kept anonymous and confidential. Your name will not be linked to the responses when we present our findings. If it is acceptable to you, I would like to tape our conversation today. This helps me go back to recall everything you said. Is it okay if I tape our conversation?

As this interview has open-ended questions, it could take about 15 minutes, is that all right?

Do you have any questions before we begin?

1. Please share with me your experience during your (home) hospital admission.
   1. Could you discuss your experiences with the physician(s)? (Did you feel comfortable calling the physician after hours or you were hesitant to do so?)
   2. Could you discuss your experiences with the nurses?
   3. How comfortable were you during your admission?
      1. Consider probing on room, bed, food, sleep, activity, pain, connection with family/friends (Did you feel that you put an extra burden on your family?), connection with care team. (Did you feel you had a better/ more personal relation with your physician/ nurse?)
   4. How safe did you feel during your admission?
      1. Did it make you feel safer knowing that it was the same physician and nurse on call 24 hours during your admission?
2. Could you describe what went smoothly during your (home) hospital admission?
3. Could you describe what difficulties you came across during your (home) hospital admission?
   1. How could the (home) hospital team have made that better for you?
4. What surprises were there during your (home) hospital admission? Can you talk a little about those?
5. Could you describe what improvements to (home) hospital could be made?
6. If heaven-forbid you had to be hospitalized again and you could choose home hospital or regular hospital, which would you choose? Why?
   1. What would you tell someone else who was making this decision?
7. Is there anything else you think we should know about your (home) hospital experience?
   1. For the patient who has a caregiver: How was your traditional caregiver affected by your hospitalization?
      1. Followup: more/less burdened.
   2. For the patient who is the caregiver: How were you able to cope with your illness during hospitalization?

Thank you very much for your time. If you have any further comments or questions, please reach out to the study coordinators.
