# Supplementary material for: Effect of hospital-at-home vs. traditional brick-and-mortar hospital care in acutely ill adults: study protocol for a pragmatic randomized controlled trial
Source: Trials. 2022 Jun 16;23:503. doi: 10.1186/s13063-022-06430-6 (PMC9201794; doi:10.1186/s13063-022-06430-6)
Supplement: Supplementary file 7 — Additional file 7. Clinician and Staff Interview Guide [file 13063_2022_6430_MOESM7_ESM.docx]

**Clinician and Staff Interview Guide**

*[Work through the consent process]*

*Today we’d like to talk with you about the Advance Care at Home (ACH) model. We may ask you some specific questions about different aspects of ACH, but we’d like to start by hearing about your general experiences so far with implementing the model into practice.*

*Before we begin, we would like to record this interview to assist with the accuracy of our notes. Do we have your permission to record? (Y/N)*

**Background**

Before we begin it would be helpful to get a sense of your background.

1. What is your role on the ACH care team?
2. Prior to joining the ACH team, did you have previous experience with home health delivery and/or remote care delivery?
3. What has your experience been working in a inpatient setting?

**Perceptions of ACH**

1. What are your overall impressions of the ACH model?
   1. How would you describe the goals of the program?
   2. What do you see as the benefits to implementing this model? *[e.g. to patients, providers, payers, etc.]*
2. Describe what it has been like to implement the ACH model into practice.
3. What elements of the model would you say we have excelled in or implemented successfully?
4. What are some of the areas where we have struggled to implement or elements of the model that have been difficult to operationalize?
5. What do you think might be contributing to some of the challenges you are experiencing?
   1. Do you see any specific clinic, facility, or system-level policies that make it difficult to implement the model? *[e.g. eligibility criteria, clinical workflow, payment/reimbursement structure, staffing levels]*
6. How have you addressed or overcome some of the challenges that you’ve experienced?
7. Have you needed to make any adaptations to ACH model to fit a specific circumstance?
8. What are your impressions of the ACH model relative to in hospital care?
   1. What do you see as some of the critical factors for an acute care delivery model like this?
   2. In what ways does the in-home setting impact
      1. the way care is delivered—Are there ways care is facilitated or hindered by the ACH model?
      2. care team function—Are there ways the teamwork facilitated or hindered by the ACH model?
      3. Patient-clinician interaction—Are there ways communication is facilitated or hindered by the ACH model?
9. As Mayo considers rolling out this model more broadly, what recommendations or advice would you have for those who are developing the model and leading efforts to evaluate it in the practice?
   1. Are there things leadership needs to keep in mind when expanding this program out on a larger scale?

**Closing Thoughts**

1. Is there anything else you would like to add that you believe is important to consider or be mindful of moving forward?
