# Supplementary material for: Effect of hospital-at-home vs. traditional brick-and-mortar hospital care in acutely ill adults: study protocol for a pragmatic randomized controlled trial
Source: Trials. 2022 Jun 16;23:503. doi: 10.1186/s13063-022-06430-6 (PMC9201794; doi:10.1186/s13063-022-06430-6)
Supplement: Supplementary file 8 — Additional file 8. Patient 30-Day Follow-Up [file 13063_2022_6430_MOESM8_ESM.docx]

**Patient 30-Day Follow-Up**

Study ID:

**Did you visit the Emergency Department (ED) between [start of follow up] and [30 days]?**

Yes 🡪 Date ____/____/________

**Were you transferred to the Intensive Care Unit (ICU) during your ED stay?**

**stay?**

Yes

No

No

**Were you re-admitted to the hospital between [start of follow up] and [30 days]?**

Yes 🡪 Date ____/____/________

**Were you transferred to the Intensive Care Unit (ICU) during your re-admission?**

**?**

Yes

No

No

**Did you fall between [start of follow up] and [30 days]?**

Yes 🡪 **Did your fall result in an injury?** Yes / No

No

**Did a medication error occur between [start of follow up] and [30 days]?**

Yes 🡪 Date ____/____/________

**Did you receive treatment for the medication error?**

Yes 🡪 Place treatment was received: ___________________

No

Unsure

**If a participant has a documented ED visit, re-admission, fall or medication error in the medical record, confirm the event and date with the participant.*

**Did you receive care outside of Mayo Clinic between [start of follow up] and [30 days]?**

Yes

**Let participant know we will be sending an authorization form to obtain copies of external records from the healthcare organization they received care at.*

No

**EQ-5D**

**Patient Experience Questions**

**1. In general, how satisfied are you with your recent (home) hospital experience?**

| Not at all satisfied | Slightly satisfied | Moderately satisfied | Very satisfied | Extremely satisfied |
| --- | --- | --- | --- | --- |
| 1 | 2 | 3 | 4 | 5 |

**2. How comfortable were you during the (home) hospital admission?**

| Not at all comfortable | Slightly comfortable | Moderately comfortable | Very comfortable | Extremely comfortable |
| --- | --- | --- | --- | --- |
| 1 | 2 | 3 | 4 | 5 |

**3. How safe did you feel during the (home) hospital admission?**

| Not at all safe | Slightly safe | Moderately safe | Very safe | Extremely safe |
| --- | --- | --- | --- | --- |
| 1 | 2 | 3 | 4 | 5 |

**4. Please provide any information that you think went particularly well or poorly to help us make improvements in the future.**

**Thank them for their time and ask their permission to be contacted to participate in an interview with a member of the study team in the future.*
