# Supplementary material for: Effect of hospital-at-home vs. traditional brick-and-mortar hospital care in acutely ill adults: study protocol for a pragmatic randomized controlled trial
Source: Trials. 2022 Jun 16;23:503. doi: 10.1186/s13063-022-06430-6 (PMC9201794; doi:10.1186/s13063-022-06430-6)
Supplement: Supplementary file 9 — Additional file 9. ACH DSMB Charter [file 13063_2022_6430_MOESM9_ESM.docx]

## DSMB Charter

## Title: Effect of Advanced Care at Home vs. Traditional Brick-and-Mortar Hospital Care in Acutely Ill Adults: A Randomized Clinical Trial

## IRBe Application: 21-005335

## PI: Sean C. Dowdy, M.D., Elizabeth B. Habermann, Ph.D.; Xiaoxi Yao, Ph.D.

## Institution: Mayo Clinic

The Data and Safety Monitoring Board (DSMB) will act in an advisory capacity to monitor participant safety, data quality and progress.

### DSMB Responsibilities

The DSMB responsibilities are to:

Initial meeting

- Review the entire IRB-approved study protocol and the MOP, with regard to participant safety, recruitment, randomization, intervention, data management, quality control and analysis and the informed consent document.
- Recommend changes to the protocol and the informed consent form, when applicable.
- Identify the relevant data parameters and the format of the information to be regularly reported.
- Recommend participant recruitment be initiated after receipt of a satisfactory protocol. If the need for modifications to the protocol, the MOP, consent form, DSMP or any other study document is indicated by the DSMB, the DSMB will postpone its recommendation for the initiation of participant recruitment until after the receipt of a satisfactory revised protocol(s) or other study documents.

During the study meetings

- Review data related to safety, recruitment, randomization, retention, protocol adherence, trial operations, data completeness, form completion, intervention effects, gender and minority inclusion.
- Identify needs for additional data relevant to safety issues and request these data from the study investigators.
- Propose additional analyses and periodically review developing data on safety and endpoints.
- At each meeting, consider the rationale for continuation of the study, with respect to progress of randomization, retention, protocol adherence, data management, safety issues, and outcome data (if relevant) and make a recommendation for or against the trial's continuation.
- Review and make recommendations on proposed protocol changes, and/or new protocols proposed during the trial. When the DSMBs are unblinded, the Boards may recommend appointing a blinded working group of the DSMB to review the proposed protocol changes and make recommendations on whether to approve the requests.
- Provide advice on issues regarding data discrepancies found by the data auditing system or other sources.
- Review manuscripts of trial results if requested by the Board who may seek DSMB review of manuscripts reporting major outcomes prior to their submission for publication.

The DSMB will discharge itself from its duties when the study is complete.

### Membership

Membership consists of persons completely independent of the investigators who have no financial, scientific or other conflict of interest with the trial. This DSMB will consist of three members.

The DSMB includes experts in or representatives of the fields of:

- Neurology and Critical Care (William [Dave] Freeman, M.D.)
- Hospital Internal Medicine (Nancy Dawson, M.D.)
- Clinical Trials and Biostatistics (Qian Shi, Ph.D.)

Nancy Dawson, M.D. has been selected to serve as the Chairperson and is responsible for facilitating the meetings, reviewing the first draft of the meeting notes and any decision making in the case of a tie vote. The Chair is the contact people for the DSMB. The Mayo Clinic will provide the logistical management and support for the DSMB.

### Meeting Format

Meetings of the DSMB will be held regularly (e.g., monthly at the beginning of the trial and adjust frequency as needed later) at the call of the DSMB Chair. An emergency meeting of the DSMB may be called at any time by the Chair, should participant safety questions or other unanticipated problems arise.

DSMB meetings will consist of open, closed and optional executive sessions, all closed to the public because discussions may address confidential participant data. The study PI, key staff members, and DSMB members attend the **open sessions**. Discussions at these sessions focus on the review of the aggregate data, conduct and progress of the study, including participant accrual, protocol compliance, and problems encountered. Data by treatment group are not presented in the open session.

The **closed session** will be attended by the statistician and the DSMB members. The primary objective of the closed sessions is to review data by study group. To ensure participants safety and well-being, DSMBs are required to review safety data by the actual treatment group. In many instances, safety data could also be the outcome data.

If necessary, an **executive session** may be requested by the DSMB and will be attended only by voting DSMB members.

The DSMB Chair or the Principal Investigator will prepare the meeting agenda that usually includes the following:

1. Welcome and introduction – study team, DSMB members
2. Open session (review study protocol and its amendments, consent form, open study report, etc.) - study team, DSMB members
3. Closed session (review closed session report, including unblinded data, etc.) – unblinded study statistician, DSMB members
4. Executive session (optional, upon DSMB request) – DSMB members only
5. Debriefing (optional, upon DSMB request, time permitting) - study team, DSMB members

The DSMB may modify its processes and procedures at any time with the discussion and approval of the PIs.

### Meeting Materials

DSMB interim report templates developed by the study staff for both the open and closed sessions and plans for interim analyses will be reviewed and either approved at the initial DSMB meeting or changes requested. Upon DSMB request, reports could be modified at any time during the study.

**Part 1 - Open Session Reports:**

Open session reports will include administrative reports that describe participants screened, enrolled, completed, and discontinued, as well as baseline characteristics of the study population. Other general information on study status may also be presented. Listings of adverse events and serious adverse events as well as any other information requested by the DSMB may also be in the open session report, but none of the data will be presented in an unblinded manner.

**Part 2 – Closed Session Report:**

Closed session reports will present the same information as presented in the open session but by unblinded treatment group.

### Reports from the DSMB

A report containing the recommendations for continuation or modification of the study will be prepared by the DSMB. The draft report will be sent to the DSMB members for review and approval not later than three weeks after the meeting. Once approved by the DSMB members, the DSMB chair will forward the DSMB recommendations to the Principal Investigator. It is the responsibility of the Principal Investigator to distribute the DSMB recommendation to all co-investigators and to ensure that copies are submitted to the IRB that reviewed and approved the study documents.

As it stated above, each meeting must include a recommendation to continue the study made by a formal DSMB majority or unanimous vote. Should the DSMB decide to issue a termination recommendation, the full vote of the DSMB is required. In the event of a split vote, majority vote will rule and a minority report should be appended. The DSMB Chair provides the tiebreaking vote in the event of a 50-50 split vote.

A recommendation to terminate the study may be made by the DSMB at any time by majority vote. If this recommendation was made during the DSMB’s Executive session, the Chair should inform the PI about the decision.

### Confidentiality

All materials, discussions and proceedings of the DSMB are completely confidential. Members and other participants in DSMB meetings are expected to maintain confidentiality.

# **Charter Acknowledgement**

I confirm that I have read and understood this Charter and will work in accordance with the responsibilities and processes set forth in the Charter.

| **Name and Role** | **Signature** | **Date** |
| --- | --- | --- |
| **Nancy Dawson, M.D.**  DSMC Chairperson |  |  |
| **William [Dave] Freeman, M.D.**  DSMB Member |  |  |
| **Qian Shi, Ph.D.**  DSMB Member |  |  |

# **APPENDIX A: DSMB Roster**

Below are the Committee Members and Contacts:

| **William [Dave] Freeman, M.D.**  Professor of Neurology and Neurosurgery  Mayo Clinic  Jacksonville, Florida, USA  **Phone: 904-953-6869**  **Email:** [Freeman.William1@mayo.edu](mailto:Freeman.William1@mayo.edu) | **Qian Shi, Ph.D.**  Professor of Biostatistics and Oncology  Mayo Clinic  Rochester, Minnesota, USA.  **Phone: 1 (507) 538-4340**  **Email:** [Shi.Qian2@mayo.edu](mailto:Shi.Qian2@mayo.edu) |
| --- | --- |
| **Nancy Dawson, M.D.**  Associate Professor of Medicine  Mayo Clinic  Jacksonville, Florida, USA  **Phone: 1 (904) 956-3032**  **E-mail:** [Dawson.Nancy11@mayo.edu](mailto:Dawson.Nancy11@mayo.edu) |  |

After the charter is finalized, the roster may be updated with a new version date, while the charter version date remains the same. The charter only requires a new version date and re-signature if charter processes change.

# **APPENDIX B: DSMB Closed Meeting Minutes Template**

**Closed / Executive Meeting Minutes for Data Review Meeting #X**

On DD/MMM/YYYY the DSMB met via teleconference to review provided data reports for the study with a data cut-off date of DD/MMM/YYYY.

Attendees: Chairperson: **Nancy Dawson, M.D.**

DSMC Members: **William [Dave] Freeman, M.D.; Qian Shi, Ph.D.**

Unblinded Biostatistician: **Andrew Dodge, M.S.**

| **Closed Session Presentation and Discussion: *[record the course of the evaluation /discussion comment of feedback regarding the data, observations, questions/clarifications by Unblinded or External Independent Biostatistician and concerns. Capture any dissenting opinions if not unanimous.]*** |
| --- |
|  |
| **Recommendations:**  (e.g. recommendations to sponsor such as the study may continue/ be amended/ be suspended be terminate and further comments as needed) |
|  |

Signature: __________________________________ Date: ______________________

Nancy Dawson, M.D.

DSMC Chairperson

# **APPENDIX C: DSMB Recommendation Form Template**

**Data and Safety Monitoring Committee (DSMC) Recommendation Form for Data Review Meeting #X**

**From:** Nancy Dawson, M.D., DSMB Chairperson

**To:**

On DD/MMM/YYYY the DSMB met via teleconference to review provided data reports for the study with a data cut-off date of DD/MMM/YYYY.

Attendees: Chairperson: **Nancy Dawson, M.D.**

DSMC Members: **William [Dave] Freeman, M.D.; Qian Shi, Ph.D.**

**Recommendations:**

|  |
| --- |
|  |
|  |
|  |

**Comments:**

|  |
| --- |
|  |
|  |
|  |
|  |

Signature: __________________________________ Date: ______________________

Nancy Dawson, M.D.

DSMC Chairperson
